# Modeling Glial Contributions to Seizures and Epileptogenesis: Cation-Chloride Cotransporters in *Drosophila melanogaster*

**DOI:** 10.1371/journal.pone.0101117

**Published:** 2014-06-27

**Authors:** Zeid M. Rusan, Olivia A. Kingsford, Mark A. Tanouye

**Affiliations:** 1 Department of Molecular and Cell Biology, University of California, Berkeley, California, United States of America; 2 Department of Environmental Science, Policy and Management, University of California, Berkeley, California, United States of America; 3 Helen Wills Neuroscience Institute, University of California, Berkeley, California, United States of America; Columbia University, United States of America

## Abstract

Flies carrying a *kcc* loss-of-function mutation are more seizure-susceptible than wild-type flies. The *kcc* gene is the highly conserved *Drosophila melanogaster* ortholog of K^+^/Cl^−^ cotransporter genes thought to be expressed in all animal cell types. Here, we examined the spatial and temporal requirements for *kcc* loss-of-function to modify seizure-susceptibility in flies. Targeted RNA interference (RNAi) of *kcc* in various sets of neurons was sufficient to induce severe seizure-sensitivity. Interestingly, *kcc* RNAi in glia was particularly effective in causing seizure-sensitivity. Knockdown of *kcc* in glia or neurons during development caused a reduction in seizure induction threshold, cell swelling, and brain volume increase in 24–48 hour old adult flies. Third instar larval peripheral nerves were enlarged when *kcc* RNAi was expressed in neurons or glia. Results suggest that a threshold of K^+^/Cl^−^ cotransport dysfunction in the nervous system during development is an important determinant of seizure-susceptibility in *Drosophila*. The findings presented are the first attributing a causative role for glial cation-chloride cotransporters in seizures and epileptogenesis. The importance of elucidating glial cell contributions to seizure disorders and the utility of *Drosophila* models is discussed.

## Introduction

Glial cells are proposed to be important players in seizure disorders because of their critical role in maintaining extracellular ionic homeostasis in the nervous system [1]–. Glial contributions to seizures, however, have not been studied as well as neuronal and synaptic signaling mechanisms. In this study, we examined cation-chloride cotransporter (CCC) function separately in neurons and in glia, and showed that a loss of CCC function in either cell class causes seizure-sensitivity in *Drosophila*.

Nine CCCs comprise the SLC12 family of transmembrane proteins (SLC12A1–A9). Evolutionarily ancient, these solute carriers are described for vertebrates, arthropods, worms, plants, fungi, and bacteria [6]–[9]. CCCs are symporters transporting Cl^−^ ions together with Na^+^ and/or K^+^ ions across the plasma membrane. The Na^+^-coupled CCCs (SLC12A1-3 or NKCC2, NKCC1, and NCC in vertebrates) utilize the large inwardly-directed electrochemical gradient for Na^+^ to transport Cl^−^ into the cell. K^+^/Cl^−^ cotransporters (SLC12A4-7 or KCC1-4 in vertebrates) mainly transport K^+^ and Cl^−^ out of the cell utilizing the electrochemical gradient for K^+^.

CCCs are involved in a variety of physiological mechanisms in humans and experimental models [10], [11]. CCCs play cell-type specific roles in the control of several fundamental processes including cell volume homeostasis, cell migration, neural circuit development and neuronal excitability [12], [13]. For example, CCCs regulate cell volume with cell swelling countered by an efflux of Cl^−^, K^+^, and water, mediated by KCC3 and ion channels [14]–[16]. Cell shrinkage is countered by an influx of Cl^−^, Na^+^, K^+^ and water, mediated by NKCC1 and ion exchangers [17], [18]. All cells tightly regulate their size and shape because even minor changes can be catastrophic to the myriad of cell and organ functions [19]–[21].

In *Drosophila*, there are five CCC genes: *kcc (kazachoc)*, an ortholog of vertebrate *KCC1*-*4*; *ncc69*, an ortholog of vertebrate *NKCC1* and *NKCC2*; and three less well-understood members (*CG12773*, *CG31547* and *CG10413*) [22]–[27]. Partial loss of *kcc* function causes seizure-sensitivity, in part via abnormal excitatory GABAergic signaling due to intracellular neuronal Cl^−^ misregulation [24]. The results for *Drosophila* resemble those of vertebrates that implicate excitatory GABAergic signaling in the genesis of neonatal seizures, temporal lobe epilepsy, and seizures occurring after ischemic-hypoxic insult [11], [20], [28]. Here, we showed that seizure-sensitivity due to *kcc* loss-of-function in *Drosophila* is more complex than previously described.

In the present experiments, CCC loss-of-function flies were generated by ectopic spatial and temporal restriction of RNA interference (RNAi) transgene expression. Seizure-sensitivity was observed in animals with *kcc* loss-of-function in either glia or neurons. *ncc69* loss-of-function in glia, but not neurons, caused seizure-sensitivity. These neurological phenotypes correlated with cell and tissue volume abnormalities in larvae and adults.

## Materials and Methods

### Fly Strains

The genotypes of the *Drosophila* strains used in this study and their sources are listed in Table S1. Flies were maintained on standard cornmeal-molasses medium at 25°C in a humidified incubator unless otherwise indicated. The *kcc^DHS1^* mutation is a 13 base pair insertion in intron 11 of *kcc (kazachoc)*. The mutation leads to an approximate two-fold reduction in *kcc* transcript as determined previously by RT-PCR; and an approximate four-fold reduction in Kcc protein as determined by Western blot analysis [24]. Genetically, *kcc^DHS1^* behaves as a hypomorph [24]. The *kcc^EY08304^* embryonic lethal mutation is a P-element insertion at 2R:19,812,398, upstream of the sequence encoding the C-terminus tail predicted to be recognized by the Kcc antibody used in this study (Fig. S1). The *kcc^Ad4^* embryonic lethal mutation is not molecularly mapped, but it fails to complement *kcc^EY08304^* and is apparently a null mutation (see [24] and Fig. S1). Genotypes were constructed by standard *Drosophila* crossing schemes using common strains with balancer chromosomes as needed.

### Behavioral Assays

Behavioral seizure-like activity, or bang-sensitive (BS) paralysis, was quantified as described previously [29]. Briefly, flies were collected <1 day post-eclosion then placed in a 25°C humidified incubator to recover from CO_2_–induced anesthesia until tested for BS paralysis the following day. For scoring BS paralysis, 15 or fewer individual flies were tested per vial. Mechanical stimulations, or “bangs”, were performed on vials using a VWR vortexer set to maximum speed for 10 s. Pools of data were then combined for each genotype (n>100 flies for each) to yield a final measure of %BS paralysis at 24–48 h-old. Movies of BS paralysis were acquired using a mobile cellular device and edited in Windows Live Movie Maker. Flies kept at 30°C for Temporal and Regional Gene Expression Targeting [30] experiments were moved to fresh vials after each BS paralysis test due to deterioration of vial conditions.

### Electrophysiology

*in vivo* electrophysiological assays were from the adult giant fiber system [31]. Seizure threshold tests were described previously [29], [32]–[34]. For mounting, the fly was suctioned onto a hypodermic needle attached to a vacuum line, then stabilized in dental wax on a glass microscope slide. Stimulating and ground electrodes were uninsulated tungsten. Recording electrodes were glass micropipettes filled with 3 M KCl. Seizure-like activity was evoked by high-frequency electrical brain stimulation (0.5 ms pulses at 200 Hz for 300 ms) of varying voltage and monitored by dorsal longitudinal muscle recordings as described previously [29], [33], [34]. Flies were given a maximum of four high-frequency stimuli total, with 7–10 minutes of rest between each stimulus before being discarded. The giant fiber circuit was monitored continuously as a proxy for holobrain function. Evoked seizures were identified by the occurrence of aberrant high-frequency dorsal longitudinal muscle activity and subsequent failure of giant fiber stimulation to elicit a muscle response, indicating chemical synapse failure [29]. Grass 44S stimulus duration was controlled by a Molecular Devices Digidata 1440a commanded by custom protocols implemented in Strathclyde Electrophysiology Software, WinWCP V4.5.8. Pulse width, stimulation frequency, and voltage settings on the stimulator were set manually. Recordings were led to a WPI Electro 705 preamplifier and digitized using the Digidata 1440a with 50 Hz sampling. n>10 animals recorded for each genotype tested.

### Histology

Whole mount *Drosophila* embryos were stained following Bossing's whole-mount protocol (Abcam). Third instar larvae were filleted in hemolymph-like solution [35] then stained according to standard procedures. Immuno-staining of 24–48 h-old adult brains was performed as described previously [36]. Kcc antibody is rabbit polyclonal anti-Kcc (Pacific Immunology, Ramona, CA). The antibody is predicted to specifically recognize all seven *Drosophila melanogaster* Kcc isoforms (Kcc-A, -B, -C, -D, -E, -F, and -G) at their identical C-termini (RGGGREVITIYS) (BLAST; National Institutes of Health). Control experiments with *kcc* deletion mutations in embryos show antibody specificity to Kcc protein (Fig. S1) consistent with previous Western blot analysis [24]. Other primary antibodies used were: Ab13970 chicken polyclonal anti-GFP (1∶1000; Abcam), nc82 mouse anti-bruchpilot (1∶500; Developmental Studies Hybridoma Bank, U Iowa) and anti-HRP-Cy3 (1∶100; Invitrogen). Secondary antibodies were IgG (H+L) Alexa Fluor 488 goat anti-chicken (1∶1000; Jackson Immunoresearch), Alexa Fluor 546 goat anti-mouse (1∶1000; Life technologies), and Alexa Fluor 647 goat anti-rabbit (1∶1000; Life technologies).

### Imaging and Quantification

Images were acquired using the following confocal microscopes and software: Zeiss LSM 5-Live with LSM 5 software, and Zeiss LSM 780 with ZEN software. Cross-channel bleed-through for each tissue type was negligible as determined by single antibody staining and ZEN software linear unmixing (data not shown). Fluorescent micrographs were processed using ImageJ software (National Institute of Health). Embryo Kcc fluorescence intensities were averages of internal regions defined by perimeter signal for each genotype, after background subtraction. Calyx Kcc fluorescence intensities were averages of regions of interest defined by the GFP+ channel for each genotype in three slices (anterior, center, and posterior of calyx), after background subtraction. Mean Kcc fluorescence intensities of embryos and adults were normalized to control genotype intensities. Adult brain size was approximated as the volume of an ellipsoid with semi-axes along the dorsal-ventral axis (top to center of brain along the midline in the central optical slice), the anterior-posterior axis (half of encompassing Z-stack size), and the lateral-medial axis (lateral border of optic lobe to the midline in the central optical slice). Volume estimation of neuron somata (assumed spherical) was achieved by measuring the longest observable diameter for each cell. Quantification of larval peripheral nerve average cross-sectional area was performed by averaging fourteen evenly-distributed diameter measurements at the center-most GFP channel optical slice along 500 µm of each nerve beginning below the ventral nerve cord (assuming each nerve was a cylinder). Peripheral nerve sections with glial cell nuclei were excluded from measurements. Statistical tests were unpaired two-sample Student's *t*-tests. Confocal slice and Z-stack projection sizes were noted in figure legends. Image files were imported into Adobe Illustrator CS6 for figure composition.

## Results

### Kcc found in glial and neuronal cell membranes

**Figure 1:**
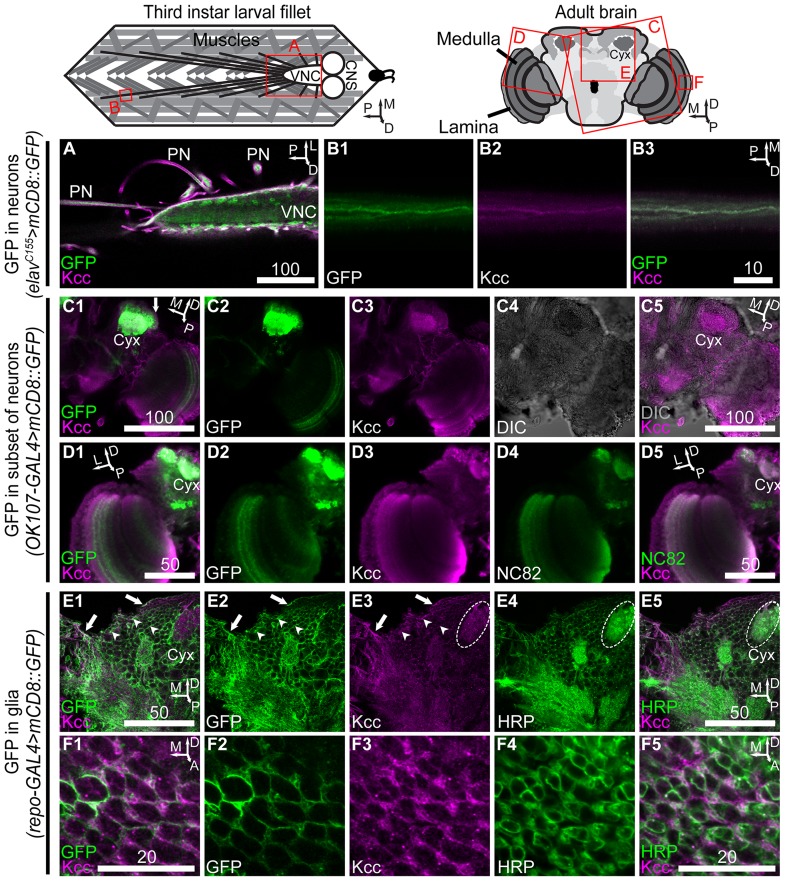
Immunohistochemistry with confocal fluorescence microscopy reveals neuronal and glial Kcc in larvae and adults. (**A**) Larval nervous system Kcc in a 0.75 µm confocal slice is seen surrounding the ventral nerve cord (VNC) and peripheral nerves (PNs), and colocalizing with neuronal somata on the periphery of the VNC. CNS: central nervous system. High-magnification PN in a 2 µm slice reveals membrane-bound GFP of neuronal processes (**B1**) and Kcc (**B2**) surrounding and colocalizing with the processes (**B3**). Adult posterior brain Kcc in a 0.5 µm slice (**C3**) depicts the general localization pattern observed in several regions; Kcc is widespread and salient around cell somata (**C4**) and on brain surface/edges (**C5**). Neuronal GFP in mushroom body somata and calyx membranes (**C2**) colocalizes with Kcc (**C1**), confirming that Kcc is in central brain neuropile and ubiquitous. Cyx: mushroom body calyx. Optic lobe Kcc in 0.5 µm slice (**D3**) conformed to the central brain pattern of neuronal membrane (**D2**) colocalization (**D1**). Optic medulla NC82 (**D4**), a neuropile marker, colocalized with Kcc (**D5**). Posterior brain Kcc in a 0.5 µm slice (**E3**) in animals expressing glial membrane-bound GFP (**E2**) colocalizes with GFP significantly (**E1**), probably in cortex (arrow heads) and surface (arrows) glia. Neuronal membrane (**E4**) colocalization with Kcc (**E5**) in the same slice is shown for comparison. High magnification micrographs of the optic lamina showed that Kcc in a 0.5 µm slice (**F3**) is present in glial (**F2**) and neuronal (**F4**) membranes of this brain structure (**F1**), (**F5**). Scale bars are in microns. For orientation, A: anterior, D: dorsal, L: lateral, M: medial, P: posterior.

Kcc protein was shown previously to be expressed throughout the adult brain of wild-type *Drosophila* with Western blot analysis showing a loss of immunoreactivity in *kcc* mutants [24]. In *kcc^DHS1^*, a loss of Kcc protein causes seizure-sensitivity [24]; *kcc^DHS1^* seizure-sensitivity can be rescued by supplying Kcc back to neurons or back to glia [25]. Here, we extend these observations of Kcc immunoreactivity with a Kcc-specific antibody (Figure S1) and confocal fluorescence microscopy to show expression is also present in the larval nervous system (Fig. 1). We find that both larval and adult nervous system staining shows Kcc in glial cells in addition to neurons.

In third instar larvae, prominent Kcc staining is present surrounding the ventral nerve cord and peripheral nerves, as well as in neuronal cell bodies of the ventral nerve cord (Fig. 1A). High-magnification images of peripheral nerve displayed robust GFP signal in sensory and motor neuron axons of animals expressing membrane-bound GFP in all neurons (Fig. 1B1). Kcc protein is present throughout the nerve (Fig. 1B2), and colocalizes with neuronal GFP (Fig. 1B3). Strong Kcc immunoreactivity was present ensheathing the peripheral nerve that appeared to terminate before sensory neuron somata (data not shown). Taken together, our interpretation is that most of the Kcc at the periphery is in subperineurial glia (SPG) cells, surface blood-brain barrier glia which are thought to ensheath peripheral nerves and terminate before the somatic and synaptic regions. Peripheral Kcc may in part correspond to that of a second type of surface glia, the perineurial glia (PG), ensheathing SPG cells [35].

In the adult brain, Kcc immunoreactivity was present in glial, as well as neuronal cell types. For example, Fig. 1C1–1C5 depicts the general pattern of staining in the adult posterior brain with Kcc labeled by anti-Kcc (Fig. 1C3) and neuronal membranes labeled with GFP (Fig. 1C2). Abundant neuronal Kcc was apparent from colocalization of Kcc and GFP in mushroom body neuronal cell bodies and neuropile (Fig. 1C1; mushroom body calyx, Cyx). A layer of non-neuronal Kcc expression appeared to be surrounding the mushroom body cell bodies (Fig. 1C1; arrow). We interpret this Kcc expression as belonging to PG and/or SPG cells, ensheathing the central nervous system [36]. This general arrangement of staining was found in many brain regions: colocalization of neuronal and Kcc staining in neuronal cell bodies and neuropile with non-neuronal Kcc expression ensheathing cell body regions (see below and data not shown). DIC image of the same optical slice showed light granular regions of cell bodies, and dark regions of neuropile (Fig. 1C4). Colocalization of cell bodies was often associated with the undulated Kcc patterns found in adult central brain (Fig. 1C5). Similarly, in the optic medulla, membrane-bound GFP was expressed in a subset of neurons (Fig. 1D2) and Kcc signal in the same optical slice (Fig. 1D3) colocalized with GFP (Fig. 1D1). Neuropile staining in the same medulla region (Fig. 1D4) showed robust colocalization with Kcc (Fig. 1D5). Kcc interlacing the medulla neuropile and surrounding cell bodies was observed as well (data not shown), most consistent with expression in glial membranes [37].

To explore nervous system Kcc expression further, specimens were prepared with triple labeling of glial cell membrane, neuronal membrane, and Kcc protein (Fig. 1E1–1F5). Imaging of the posterior central brain, for example, further confirmed the presence of Kcc in glial cells, apparently in surface glia and cortex glia (arrows and arrowheads, respectively, Fig. 1E1–1E3). In some instances, we were also able to infer likely Kcc-positive glial subtypes by manually tracing structures in GFP/HRP and Kcc/HRP merge Z-stacks, and basing identification on stereotyped anatomical relationships between glial subtypes and neurons ([36] and Fig. 1E4,1E5). High-magnification images of the optic lamina exposed a non-uniform distribution of Kcc in glia and neurons (Fig. 1F1–1F5). Neuronal processes contained Kcc foci (Fig. 1F5), while glial Kcc revealed in this region appear to be astrocyte-like glial processes mingling with synapses (Fig. 1F1). In summary, brain Kcc was membrane bound and found in both neurons and glia. Neuronal cell bodies, cortex glia, and surface glia all appear to have prominent Kcc immunoreactivity.

### Neuronal expression of *kcc* RNAi transgenes causes behavioral seizure-like activity

Loss of *Drosophila* K^+^/Cl^−^ cotransporter function, such as in the *kcc^DHS1^* hypomorphic mutant, raises seizure-susceptibility through a reduction in *kcc* expression. The *kcc^DHS1^* mutant displays seizure-sensitivity as evidenced by a lowered threshold to evoked electrophysiologically recorded seizure-like activity, and bang-sensitive behavioral seizure-like activity and paralysis (BS paralysis) [24], [25]. Here, we dissected spatial aspects of induced seizure-sensitivity using different GAL4 drivers and UAS-kcc-RNAi transgenes to generate *kcc* loss-of-function in subsets of cells [38]. Briefly, flies with promoter-determined GAL4 transcription factor expression (driver) were crossed to flies carrying a UAS-kcc-RNAi transgene (*kcc* RNAi under control of Upstream Activation Sequence; UAS) to drive promoter-determined *kcc* knockdown in F1 progeny (i.e. *promoter-GAL4>UAS-kcc-RNAi* progeny). We tested a total of 64 different GAL4 drivers in combination with two different *kcc* RNAi transgenes for BS paralysis. We found that this was a robust method of inducing seizure-sensitivity, particularly using UAS-kcc-RNAi-B (Bloomington stock center #34584, referred to hereafter as kcc-RNAi-B), which appeared to be more effective than UAS-kcc-RNAi-V (Vienna Drosophila RNAi Center #101742, referred to hereafter as kcc-RNAi-V) (Table S2). Twenty-eight of the 64 GAL4 drivers tested induced lethal and/or BS paralysis phenotypes. The other 36 GAL4 drivers gave rise to viable flies without BS paralysis phenotypes.

**Figure 2:**
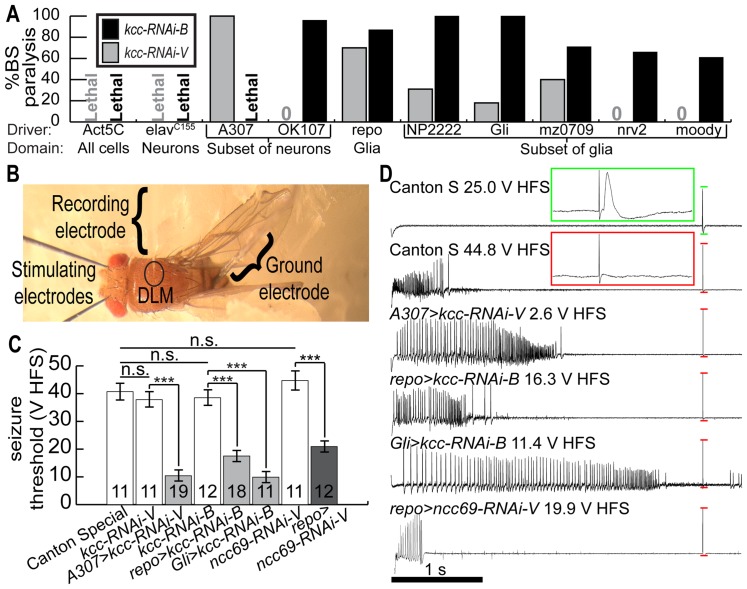
Reducing *kcc* expression by RNAi causes behavioral and electrophysiologically-recorded seizure-like activity. (**A**) Quantification of behavioral seizure-sensitivity for select GAL4/UAS genotypes, expressed as %bang-sensitive (%BS) paralysis on the y-axis, using two different UAS-kcc-RNAi transgenes. The GAL4 driver and expression domain for each genotype is shown on the x-axis. kcc-RNAi-B (black bars/text) is more effective than kcc-RNAi-V (grey bars/text) with respect to causing %BS paralysis and lethality phenotypes. (**B**) *in vivo* stimulation and recording from the giant fiber circuit of a fly mounted in dental wax for quantifying thresholds to evoked seizure-like activity. (**C**) High-frequency stimulus (200 Hz for 300 ms) seizure-like activity voltage thresholds, in volts high-frequency stimulus (V HFS), for select test and control genotypes. N-values are as noted for each genotype. Error bars are S.E.M. and significance for Student's *t*-tests is: ***  = p<0.001; n.s.  =  not significant. RNAi expression caused reduced V HFS thresholds relative to controls, thus indicating increased seizure-sensitivity. (**D**) Representative seizure-like discharges recorded in dorsal longitudinal muscles from flies of select genotypes, as indicated. Green insert is an enlargement of the region enclosed by green lines illustrating a muscle response following a giant fiber threshold stimulus pulse (∼2 V, 0.3 ms pulse-width). Red insert is an enlargement of the region enclosed by the first pair of red lines illustrating a failure following a single giant fiber threshold stimulus pulse. Remaining pairs of red lines indicated failures following seizures in other genotypes.

Several GAL4/UAS combinations resulted in lethal phenotypes, especially using the ubiquitous driver Act5C and the pan-neuronal driver elav^c155^ with either kcc-RNAi transgene (Fig. 2A). Third instar larvae with kcc-RNAi-V driven by elav^c155^ and several other GAL4/UAS genotypes often burrowed to the bottom of their vials before perishing (data not shown). Cholinergic neuron drivers caused semi-lethality and 100% BS paralysis in escapers with kcc-RNAi-V, and produced late pupal lethality with kcc-RNAi-B (Table S2). In addition, kcc-RNAi-V flies driven with cholinergic neuron drivers are sterile and have a completely penetrant juvenile-wing phenotype identical to that of flies with perturbed CCAP neuropeptide-producing/-receptive neurons (data not shown). Taken together, these observations demonstrate further the criticality of *kcc* functions throughout the *Drosophila* nervous system.

For several neuronal GAL4 drivers, we consistently observed interesting, albeit somewhat surprising results comparing kcc-RNAi-V and kcc-RNAi-B. These GAL4 drivers were: the ellipsoid body driver, c507, the motor neuron driver, OK6, and the mushroom body drivers, 201Y, MB247, OK107, and c772: for each, the weaker kcc-RNAi-V is largely ineffective in inducing BS paralytic behavior. In contrast, in combination with the stronger kcc-RNAi-B, the mushroom body drivers induce complete (100%) or nearly complete (>90%) BS paralysis, and c507 and OK6 drivers cause lethality. These data suggest that a threshold of *kcc* RNAi in a particular neuronal population must be reached in order for phenotypes to manifest. Despite this unusual observation between the two RNAi transgenes, the mushroom body GAL4 drivers did not appear to be markedly more effective at inducing BS paralysis phenotypes in these experiments than any other neural drivers.

Examination of 49 GAL4/UAS combinations using mushroom body and other neuronal GAL4 drivers showed no obvious spatial tendencies in the induction of seizure-sensitivity by *kcc* RNAi. Drivers expressing in all or many neurons induced lethality and/or extreme BS paralysis (Fig. 2A). Drivers thought to be expressed in small sets of neurons, including GABAergic, U/CQ, and Pdf-expressing neurons, did not elicit BS paralysis in combination with either kcc-RNAi-V or kcc-RNAi-B transgene (Table S2). Non-nervous system drivers, including those with robust hemocyte, salivary gland, fat body, muscle, tracheal, and adipokinetic hormone-secreting cell expression, always generated viable flies without BS paralysis phenotypes (Table S2). Cumulatively, it appeared that phenotypes were mainly associated with the number of neurons deficient in *kcc* function, and the magnitude of this deficiency.

### BS paralysis caused by glial expression of *kcc* RNAi

Experiments using glial-specific GAL4 drivers indicate directly that loss of *kcc* function in this tissue causes seizure-sensitivity. repo-GAL4 drives expression in nearly all glia. When used to drive kcc-RNAi transgenes, we found that kcc-RNAi-V and kcc-RNAi-B flies showed substantial BS paralytic behavior: 70% and 85% BS paralysis, respectively (Fig. 2A and Movie S1). These were surprising findings, particularly since expressing *kcc* ^+^ in glial cells was not remarkably effective in rescuing *kcc^DHS1^* mutant BS phenotypes, although some rescue was observed [25].

Several glial subtypes are present in *Drosophila* [36], [37], [39], [40]. Particularly interesting are the SPG cells comprising the main blood-brain barrier cell type: pericellular diffusion being limited by septate junctions formed between the SPGs. GAL4 drivers apparently specific for cells that express septate junction genes, including SPG cells, were particularly effective at causing BS paralytic behavioral phenotypes in combination with *kcc* RNAi. Thus, Gli-GAL4 and Moody-GAL4 each driving kcc-RNAi-B in SPG gave rise to 100% and 65% BS paralysis, respectively (Fig. 2A and Movie S2). Additional phenotypes were observed in the *Gli-GAL4>kcc-RNAi-B* flies. Flies often displayed malformed wings (data not shown) and were especially sensitive to behavioral seizure-like activity. Gentle handling of the vial was sufficient to trigger seizures; this appears to be the most severely seizure-sensitive genotype that we have observed for *Drosophila* to date. Substantial (90%) BS paralysis was also observed with NP2222-GAL4 driving kcc-RNAi-B (Fig. 2A). NP2222-GAL4 is reported to drive expression in cortex glia surrounding neuronal cell bodies [41]. We also observed BS paralysis in flies with kcc-RNAi-B driven with nrv2-GAL4 (cortex and neuropile glia, 88% BS paralysis), mz709-GAL4 (ensheathing glia, 60% BS paralysis) and alrm-GAL4 (astrocyte-like glia, 22% BS paralysis) (Fig. 2A). Taken together, these results suggested that proper *kcc* function is required in glial cells, and that loss-of-function causes seizure-sensitivity. Further, within the limitations of GAL4 glial driver specificity, several different glial subtypes appeared to contribute to the seizure-sensitive phenotype due to *kcc* loss-of-function.

### Electrophysiologically recorded seizure-like activity with *kcc* RNAi

The BS paralysis phenotype is an indicator of seizure-sensitivity: flies that show a strong BS behavioral phenotype also display a reduced threshold to evoked seizure-like neuronal activity/sustained discharges in electrophysiological tests (Fig. 2B), whereas mutations that decrease behavioral bang-sensitivity raise the threshold [29], [42]. Examination of *kcc* RNAi-induced seizure-susceptibility showed that the threshold to evoked seizure-like discharges of experimental A307-GAL4 (driving in a subset of neurons [43], [44]) flies (genotype: *A307-GAL4>kcc-RNAi-V*) was about one-fourth that of wild-type and control genotypes (Fig. 2C). Thus, *A307-GAL4>kcc-RNAi-V* flies showed a low threshold of 10.7±2.1 volts high-frequency stimulus (V HFS; see Materials and Methods) whereas kcc-RNAi-V control flies had a threshold of 37.8±2.7 V HFS, comparable with wild-type Canton-S flies (40.7±2.7 V HFS). For comparison, the threshold of *A307-GAL4>kcc-RNAi-V* flies was similar to that of *kcc^DHS1^* mutant flies (13.1±4.67 V HFS, [24]).

Reducing *kcc* expression selectively in glial cells also generated flies with low thresholds for evoked seizure-like discharges (Fig. 2C), consistent with the notion that BS paralysis is a good indicator of seizure-sensitivity. Thus, *repo-GAL4>kcc-RNAi-B* flies (expressing RNAi in nearly all glia) had a low threshold of 17.5±1.5 V HFS. Thresholds were even lower for flies of the genotype *Gli-GAL4>kcc-RNAi-B* (9.9±1.9 V HFS) expressing RNAi in the SPG subset of glia. Representative traces of seizure-like discharges recorded from dorsal longitudinal muscles of the aforementioned genotypes are presented in Fig. 2D. These electrophysiological results confirmed behavioral observations indicating that reducing expression of *kcc* by RNAi in either nerve cells or glial cells increases seizure-sensitivity.

### Changes in brain anatomy observed with *kcc* RNAi

**Figure 3:**
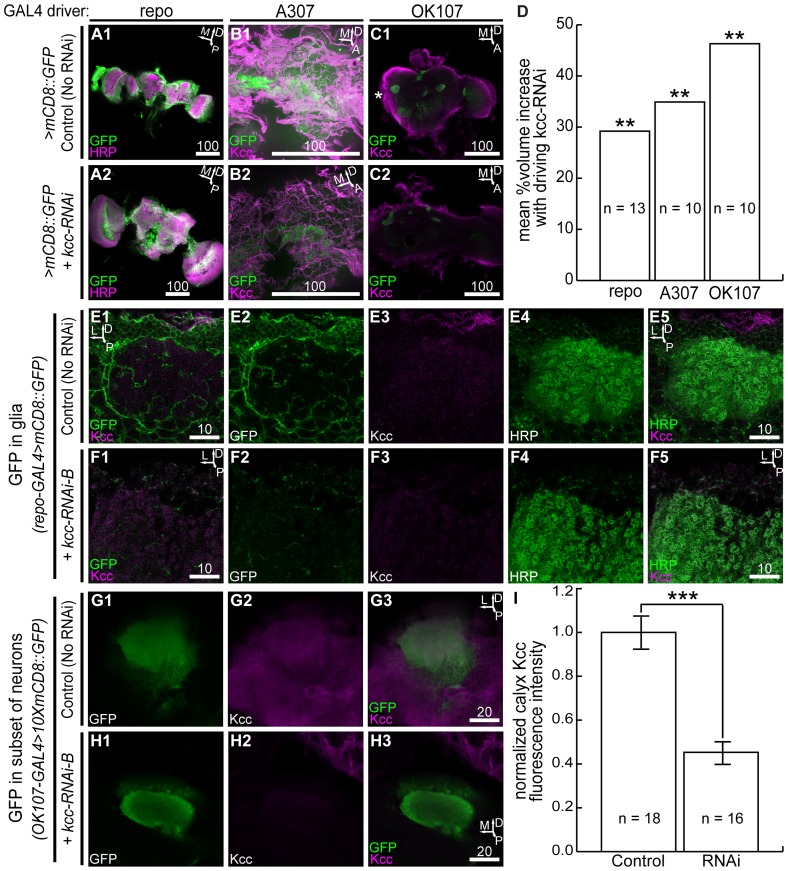
*kcc* knockdown leads to brain volume increases in 24–48 h-old adults. Shown are representative brain enlargements via *kcc* RNAi in 0.5 µm confocal slices, (**A2**), (**B2**), (**C2**), with respect to controls, (**A1**), (**B1**), (**C1**), for the repo, A307, and OK107 GAL4 drivers. *: optic lobes of test or control brains were often severed to distinguish genotypes stained in same solutions. (**D**) Quantification of mean %volume increases for test genotype brains compared to their respective controls. Expressing *kcc* RNAi in glia or neurons causes whole-brain swelling. High magnification of glia with membrane-bound GFP in the wild-type mushroom body region (**E2**) wraps and interlaces neuronal somata and the calyx neuropile (**E4**). Dorsal Kcc in this same region (**E3**) colocalizes with cortex and surface glia (**E1**) and with glomeruli of the calyx (**E5**). Glia expressing membrane-bound GFP and kcc-RNAi-B in similar mushroom body regions (**F2**) are largely absent; defined cortex glia, glia wrapping the calyx, and stereotyped surface glia are few, and thus, Kcc colocalization is reduced (**F1**). Kcc in this region (**F3**) mostly colocalizes with the neuronal calyx (**F4**) neuropile (**F5**). Neuronal expression of membrane-bound GFP in the mushroom body (**G1**) confirms Kcc localization (**G2**) in the somata and calyx neuropile (**G3**). (**H1**) *kcc* RNAi and membrane-bound GFP expression in the mushroom body caused a significant reduction of Kcc (**H2**) in the calyx (**H3**). Moreover, brain surface Kcc is further from the calyx in this loss-of-function genotype, as seen in other genotypes with neuronal *kcc* RNAi expression (data not shown). (**I**) Quantification of Kcc knockdown in the mushroom body calyx due to *kcc* RNAi expression. Significance for Student's *t*-tests is: **  = p<0.01; ***  = p<0.001. Scale bars are in microns.

Several GAL4/UAS-kcc-RNAi genotypes exhibited abnormalities in adult flies that appeared as increases in brain volume. For example, there was an approximate 1.3-fold volume increase in flies with kcc-RNAi-B driven by repo-GAL4 compared to wild-type (Fig. 3A1,3A2). Thus, estimated brain volume for experimental flies (genotype: *repo-GAL4>kcc-RNAi-B,mCD8::GFP*; volume: 5.49×10^6^±2.70×10^5^ µm^3^, n = 12) was significantly larger than for wild-type brains (4.25×10^6^±2.74×10^5^ µm^3^, n = 13; p = 0.0055) (Fig. 3D). Comparable whole-brain volume increases were observed using neuronal drivers; for example, *A307-GAL4>kcc-RNAi-V,mCD8::GFP* (Fig. 3B2), and *OK107-GAL4>kcc-RNAi-B,mCD8::GFP* (Fig. 3C2), were enlarged in comparison with brains of control genotypes (Fig. 3B1,3C1,3D).

Glial abnormalities were observed in oversized adult brains (genotype: *repo-GAL4>kcc-RNAi-B,mCD8::GFP*). Whereas, in wild-type brains mushroom body neuronal cell bodies and calyces were wrapped and interlaced with glial membranes (Fig. 3A1,3E1), these associations were largely absent in flies with glia expressing kcc-RNAi-B (Fig. 3A2,3F1). Notably, neuropile glia do not envelop the calyx when glia express kcc-RNAi-B during development (Fig. 3F1). In both control and *repo-GAL4>kcc-RNAi-B* genotypes, the neuron-enriched mushroom body calyx showed Kcc immunoreactivity (Fig. 3E5,3F5).

We frequently observed inner brain confocal image regions that appeared to be devoid of signal (GFP, Kcc or HRP) in genotypes with *kcc* RNAi expression. For example, the *repo-GAL4>kcc-RNAi-B,mCD8::GFP* (Fig. 3F1,3F5), and the *OK107-GAL4>kcc-RNAi-B,mCD8::GFP* (Fig. 3H3) calyces are surrounded by signal-devoid regions, compared to those of control genotypes (Fig. 3E1,3E5,3G3). Kcc fluorescence intensity of the *OK107-GAL4>kcc-RNAi-B,mCD8::GFP* calyx is significantly lower than that of the control calyx (Fig. 3I), however, the Kcc intensity of signal-devoid regions are even lower and comparable to intensities measured outside of the brain (data not shown).

**Figure 4:**
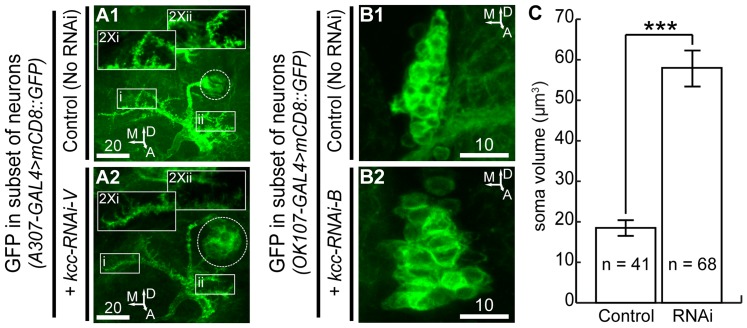
Giant fiber and lateral pace-making neurons deficient in Kcc are enlarged. (**A1**) A 34 µm Z-stack projection in animals expressing membrane-bound GFP in the giant fiber system depicts the stereotyped morphology of the wild-type giant fiber soma, axon and dendrites (insets). (**A2**) Animals expressing GFP and kcc-RNAi-V in the giant fiber system have giant fiber neurons with enlarged somata and diffuse dendrites lacking defined spines (insets), as seen in this representative 34 µm Z-stack projection. (**B1**) Wild-type lateral pace-making neurons expressing membrane-bound GFP are nearly spherical, as shown in this representative 10 µm Z-stack projection. (**B2**) Lateral pace-making neurons expressing GFP and kcc-RNAi-B are enlarged, as seen in this larger (15 µm) representative encompassing Z-stack projection. (**C**) Quantification of soma volume for control and *kcc* RNAi expressing lateral pace-making neurons. Error bars are S.E.M. and significance for Student's *t*-test is: ***  = p<0.001. Scale bars are in microns. For orientation, A: anterior, D: dorsal, L: lateral, M: medial.

The somata and processes of neurons expressing kcc-RNAi had relatively normal anatomical positions, but morphological differences were observed. Some identified cells, such as giant fiber and lateral pace-making neurons, were enlarged when expressing *kcc* RNAi transgenes (Fig. 4A2,4B2). Lateral pace-making neurons expressing kcc-RNAi-B had an average soma volume of 58.0±5.1 µm^3^ compared to 18.5±1.8 µm^3^ of controls (Fig. 4C). Diffuse and elongated dendrites lacking distinctive spines were also evident in enlarged giant fiber neurons expressing kcc-RNAi-V (Fig. 4A2; insets) compared to wild-type giant fiber neurons (Fig. 4A1; insets).

### *ncc69* RNAi in glia, but not in neurons, causes seizure-like phenotypes

Although a glial *kcc* function in *Drosophila* has not been described previously, the *Drosophila* Na^+^/K^+^/2Cl^-^ CCC homolog, *ncc69*, was shown to function in glia, regulating extracellular volume [26], [45]. Here, we examined *ncc69* loss-of-function flies for seizure-sensitivity. Two UAS-ncc69-RNAi constructs, UAS-ncc69-RNAi-B (Bloomington stock center #28682, referred to hereafter as ncc69-RNAi-B) and UAS-ncc69-RNAi-V (Vienna Drosophila RNAi Center #30000 [26], referred to hereafter as ncc69-RNAi-V), were driven with different GAL4 drivers. *ncc69* RNAi transgenes driven by repo-GAL4 in nearly all glial cells caused substantial BS paralysis (ncc69-RNAi-B 35%BS, ncc69-RNAi-V 58%BS and Movie S3). In contrast, loss of *ncc69* function in neurons caused no BS paralysis phenotypes. For example, the potent pan-neuronal driver elav^C155^ driving either kcc-RNAi-B or kcc-RNAi-V caused 0%BS paralysis. Electrophysiological tests on *repo-GAL4>ncc69-RNAi-V* flies showed a low evoked seizure-like discharge threshold, 20.9±2.2 V HFS, compared to control flies (genotype: *UAS-ncc69RNAi-V*, with no GAL4 driver; threshold: 44.7±2.7 V HFS) (Fig. 2C). A representative trace of *repo-GAL4>ncc69-RNAi-V* evoked discharges is depicted in Fig. 2D. Therefore, seizure-sensitivity by ncc69-RNAi-V paralleled volume regulation phenotypes seen with utilizing the same RNAi transgene: knockdown of *ncc69* in glia causes seizure-sensitivity (Fig. 2C) and cell volume misregulation [26], whereas, *ncc69* knockdown in neurons does not cause any detectable behavioral seizure-sensitivity (0%BS) or cell volume phenotypes [27].

### *kcc* RNAi in glia or neurons causes swelling of third instar larvae peripheral nerves

**Figure 5:**
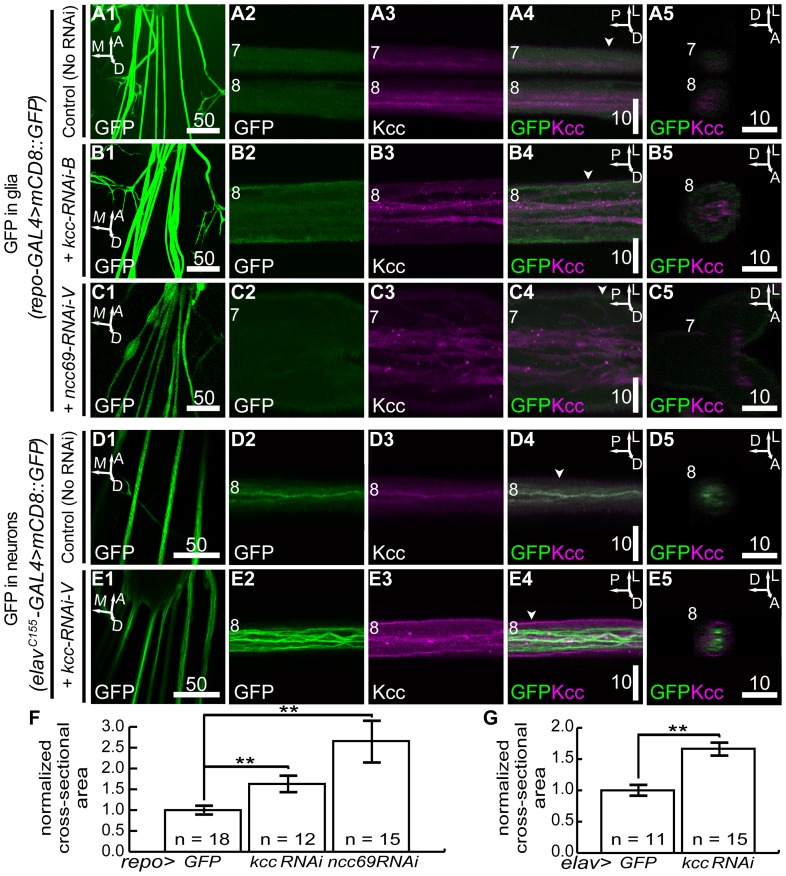
*kcc* knockdown causes swelling of third instar larval peripheral nerves. (**A1**) Membrane-bound glial GFP illuminating peripheral nerves at a low magnification 40 µm slice. (**A2**) Peripheral nerve glia GFP in a high magnification 2 µm slice colocalizes with the pervasive Kcc (**A3**) that also marks the bundled neuronal processes (**A4**). (**A5**) Orthogonal view of A4 at indicated position (A4, arrowhead). (**B1**) Glial GFP of swollen peripheral nerves in a low magnification 40 µm slice of animals also expressing glial kcc-RNAi-B. High magnification 2 µm slice of nerve glial GFP (**B2**) and Kcc (**B3**) showing whole nerve enlargement (**B4**). (**B5**) Orthogonal view of B4 at indicated position (B4, arowhead). (**C1**) Glial GFP of peripheral nerve in low magnification 40 µm slice of animals also expressing glial ncc69-RNAi-V. High magnification 2 µm slice of nerve glial GFP (**C2**) and Kcc (**C3**) showing swelling and fraying in a peripheral nerve bulge (**C4**), as shown previously [26]. (**C5**) Orthogonal view of C4 at indicated position (C4, arrowhead). (**D1**) Neuronal GFP of peripheral nerves in low magnification 40 µm slice. (**D2**) Wild-type neuronal processes in animals expressing neuronal membrane-bound GFP are tightly bundled within peripheral nerve with pervasive Kcc (**D3**). (**D4**) Kcc is within and surrounding nerve GFP+ neuronal processes of wild-type larvae. (**D5**) Orthogonal view of D4 at indicated position (D4, arrowhead). (**E1**) Neuronal GFP of peripheral nerve in low magnification 40 µm slice of animals also expressing neuronal kcc-RNAi-V. Neuronal processes within peripheral nerve (**E2**) and Kcc mostly on the surface (**E3**) do not extensively overlap (**E4**). (**E5**) Orthogonal view of E4 at indicated position (E4, arrowhead). (**F**) and (**G**) Quantification of average cross-sectional areas of peripheral nerves in control and RNAi genotypes. Error bars are S.E.M. and significance for Student's *t*-tests is: **  = p<0.01. White numbers indicate abdominal nerve number. Scale bars are in microns.

Reduced glial *ncc69* expression in third instar larvae caused abnormalities in abdominal peripheral nerves [26]. Nerves lacking Ncc69 (Na^+^/K^+^/2Cl^−^ transport function) show prominent localized swellings and defasciculation of neuronal processes, termed “fraying” [26], [27]. Peripheral nerve swelling phenotypes were observed here with glial *kcc* loss-of-function. Fig. 5 compares third instar larvae abdominal nerves of different genotypes. *repo-GAL4>kcc-RNAi-B,mCD8::GFP* larvae (expressing *kcc* RNAi and membrane-bound GFP in nearly all glia) displayed an increase in the average nerve cross-sectional area along the entire length of their nerves (Fig. 5B1,5B2,5F), without localized swelling, relative to those of control *repo-GAL4>mCD8::GFP* larvae (Fig. 5A1,5A2,5F). The *kcc* RNAi phenotypes were completely penetrant with volume increases appearing in the abdominal nerves of all animals examined (n = 12, Fig. 5F). These phenotypes were evident, but appeared to be less severe than those of nerve swellings in glial *ncc69* RNAi larvae (Fig. 5C1-5C5,5F; [26]).

Knockdown of *kcc* in neurons also caused a nerve swelling phenotype, as seen in *elav^C155^>kcc-RNAi-V,mCD8::GFP* larvae (expressing *kcc* RNAi and membrane-bound GFP in all neurons), albeit less severe than that of glial *kcc* knockdown (Fig. 5E1-5E5). Peripheral nerve size was significantly altered when compared to control *elav^C155^>mCD8::GFP* larvae (Fig. 5D1-5D5,5G). Thus, the larval CCC loss-of-function results bolstered the connection between volume regulation abnormalities and seizure-susceptibility; *kcc* or *ncc69* knockdown in glia, and *kcc* knockdown in neurons, correlated with larval volume abnormalities, adult volume abnormalities, and adult seizure-sensitivity.

## Discussion

In *Drosophila*, a combination of advanced genetic, behavior, and electrophysiology methods facilitate investigations of neurological dysfunction, especially seizure disorders. We showed here that RNAi, particularly *kcc* RNAi, is an especially effective approach for generating seizure-like phenotypes in flies. This, together with spatial and temporal control of expression, enables incisive investigations into possible mechanisms underlying seizure-susceptibility to be conducted. We found, surprisingly, that targeting *kcc* knockdown separately to either neurons or to glia induced seizure-sensitivity. Either *kcc* loss-of-function condition caused flies to exhibit robust seizure-like behaviors and electrophysiologically recorded seizure-like discharges. Immunohistochemistry showed Kcc was present in both neurons and glia at different stages of development, confirming the expectation that expression is present in both of these cell types.

Seizure-sensitivity in *kcc^DHS1^* mutants was previously shown to be mediated by *Drosophila* GABA_A_ receptors, thereby linking *kcc* loss-of-function seizures with dysfunction of the GABAergic inhibitory system [24], [25]. This suggested further that seizure-sensitivity via *kcc* dysfunction is caused by, or exacerbated by, excitatory GABA signaling due to neuronal intracellular Cl^-^ misregulation. This interpretation is similar to the mechanism thought to be responsible for seizures due to reduced KCC2 function in mice and humans [46]–[49].

Excitatory GABA signaling is an unlikely mechanism for seizure-sensitivity following *kcc* knockdown in glia. The similarity of seizure-sensitive phenotypes due to both *kcc* knockdown and *ncc69* knockdown suggests the possibility of a common mechanism, as these two transporters are closely associated in many tissues, albeit generally with reciprocal functions in terms of Cl^−^ flux [12]. Both *kcc* and *ncc69* knockdown in glia also caused larval nerve swelling [27]. There are, therefore, several salient questions related to these transporters: 1) how do Kcc and Ncc69 function in glia? 2) how does CCC loss in glia cause phenotypes of neuronal dysfunction? and 3) is there a causal relationship between nervous system phenotypes, for example, are the structural phenotypes of swelling responsible for the seizure-sensitivity phenotypes? We attempted to partially answer these questions by assessing the developmental requirement for *kcc* RNAi-related seizures using the heat-shock GAL4 and TARGET temporally-controlled expression systems [30]. Behavioral seizure-like activity was never observed in adult flies undergoing several *kcc* RNAi induction paradigms spanning multiple days, having developed as wild-type (data not shown). Thus, the questions related to CCCs in glia require more investigation to be answered.

Glial contributions to seizure-susceptibility in humans and mammalian models are not well-studied, but are believed to arise primarily from a dysfunction in ionic homeostasis, mainly a failure of glial cells to adequately buffer extracellular K^+^ (reviewed in [2]). This is a particular problem in post-traumatic models of epilepsy highlighted by studies showing that acute osmotic disruption of the blood-brain barrier causes seizures in human patients and porcine models [50]. Volume changes to neurons are also known to increase seizure-sensitivity and increase the severity of epileptiform activity (reviewed in [21]). Understanding the ways in which CCCs are involved in epilepsies may aid in the design of less toxic cures for seizures related to CCC dysfunction. The *Drosophila* experimental model for CCC loss-of-function studies can provide unique insight into glial CCC mechanisms. Although biochemical properties and loss-of-function phenotypes of glial CCCs have been explored previously *in vitro*, we are unaware of other studies exclusively targeting glial CCC functions *in vivo* besides the work presented here and that of Leiserson et al. in *Drosophila* [16], [17], [26], [51], [52]. The conservation of glial functions and the currently unmatched genetic tools for manipulating them in *Drosophila* make the fly an indispensable model for elucidating glial seizure mechanisms [41], [53].

## Supporting Information

Figure S1 **Rabbit polyclonal anti-Kcc is largely Kcc-specific.** Representative images of 20–22 h-old F1 embryos of self-crossed *kcc^Ad4^*/*Cyo*,*Act-GFP* flies (with ubiquitous cytoplasmic GFP expression in all cells) stained with anti-GFP (**A1**) and anti-Kcc (**A2**). Faint auto-fluorescence in GFP-negative embryos arising from developing gut can be seen in the GFP channel confirming that they, and other similarly scored specimens, were not unfertilized eggs. GFP-negative embryos were presumably *kcc^Ad4^*/*kcc^Ad4^*. Marginal Kcc signal was detected surrounding all embryos of any genotype suggesting a negligible degree of non-specific staining or presence of persistent maternally-deposited Kcc. (**A3**) GFP-negative embryos were almost always Kcc-negative (n = 42 out of 51). Thus, *kccAd4* appears to be a null mutation. Representative images of 20–22 h-old F1 embryos of self-crossed *kcc^EY08304^*/*Cyo*,*Act-GFP* flies stained with anti-GFP (**B1**) and anti-Kcc (**B2**). GFP-negative embryos are presumably *kcc^EY08304^*/*kcc^EY08304^*. (**B3**) GFP-negative embryos were almost always Kcc-negative (n = 28 out of 33). Thus, *kcc^EY08304^* appears to be a null mutation. (**C**) Quantification of highly significant Kcc loss in *kcc^Ad4^*/*kcc^Ad4^* mutant embryos compared to controls. (**D**) Quantification of highly significant Kcc loss in *kcc^EY08304^*/*kcc^EY08304^* mutant embryos compared to controls. Error bars are S.E.M. and significance for Student's *t*-tests is: ***  = p<0.001. Scale bars are in microns.
(TIF)

Table S1 **The genotypes and sources of the** ***Drosophila*** **strains used in this study.**
(DOC)

Table S2 **All results from the UAS-kcc-RNAi-V and UAS-kcc-RNAi-B screen for RNAi-induced behavioral seizure-like activity.**
(DOC)

Movie S1 **Behavioral seizure-like activity of three 24-48 h-old repo-** ***GAL4>kcc-RNAi-B*** **flies.**
(MP4)

Movie S2 **Behavioral seizure-like activity of eight 24-48 h-old** ***Gli-GAL4>kcc-RNAi-B*** **flies.**
(MP4)

Movie S3 **Behavioral seizure-like activity of six 24-48 h-old** ***repo-GAL4>ncc69-RNAi-V*** **flies.**
(MP4)
