# Correction: Modeling Glial Contributions to Seizures and Epileptogenesis: Cation-Chloride Cotransporters in *Drosophila melanogaster*

**DOI:** 10.1371/journal.pone.0108081

**Published:** 2014-09-05

**Authors:** The PLOS ONE Staff

There is an error in the reference range of the first sentence of the Introduction in the XML version of the article. The sentence should read: Glial cells are proposed to be important players in seizure disorders because of their critical role in maintaining extracellular ionic homeostasis in the nervous system [1]-[5].
