# Supplementary figures and images for: Modeling Glial Contributions to Seizures and Epileptogenesis: Cation-Chloride Cotransporters in Drosophila melanogaster
Source: PLoS One. 2014 Jun 27;9(6):e101117. doi: 10.1371/journal.pone.0101117 (PMC4074161; doi:10.1371/journal.pone.0101117)

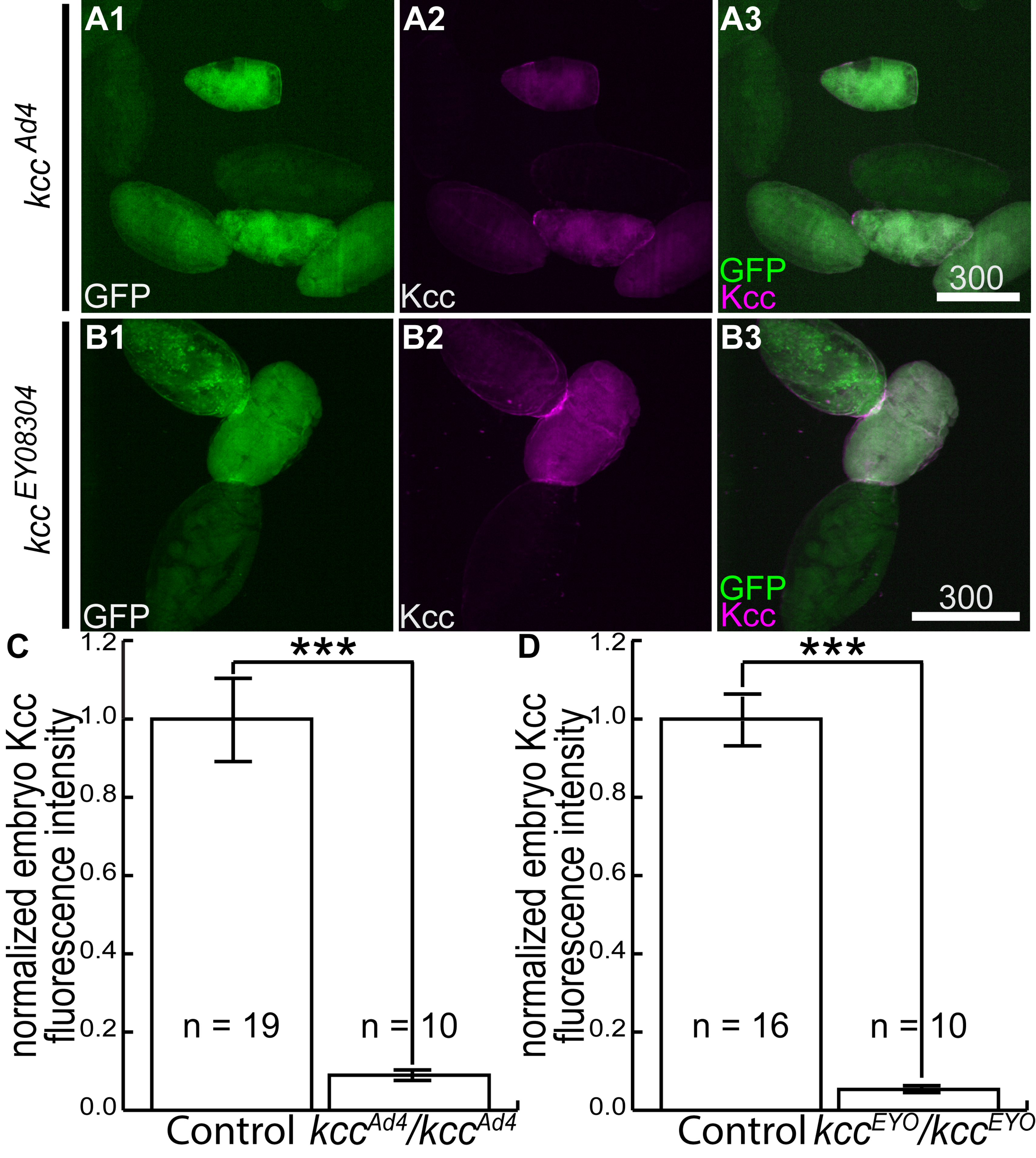

Supplement: Figure S1 — Rabbit polyclonal anti-Kcc is largely Kcc-specific. Representative images of 20–22 h-old F1 embryos of self-crossed kccAd4/Cyo,Act-GFP flies (with ubiquitous cytoplasmic GFP expression in all cells) stained with anti-GFP (A1) and anti-Kcc (A2). Faint auto-fluorescence in GFP-negative embryos arising from developing gut can be seen in the GFP channel confirming that they, and other similarly scored specimens, were not unfertilized eggs. GFP-negative embryos were presumably kccAd4/kccAd4. Marginal Kcc signal was detected surrounding all embryos of any genotype suggesting a negligible degree of non-specific staining or presence of persistent maternally-deposited Kcc. (A3) GFP-negative embryos were almost always Kcc-negative (n = 42 out of 51). Thus, kccAd4 appears to be a null mutation. Representative images of 20–22 h-old F1 embryos of self-crossed kccEY08304/Cyo,Act-GFP flies stained with anti-GFP (B1) and anti-Kcc (B2). GFP-negative embryos are presumably kccEY08304/kccEY08304. (B3) GFP-negative embryos were almost always Kcc-negative (n = 28 out of 33). Thus, kccEY08304 appears to be a null mutation. (C) Quantification of highly significant Kcc loss in kccAd4/kccAd4 mutant embryos compared to controls. (D) Quantification of highly significant Kcc loss in kccEY08304/kccEY08304 mutant embryos compared to controls. Error bars are S.E.M. and significance for Student's t-tests is: *** = p<0.001. Scale bars are in microns. (TIF) [file pone.0101117.s001.tif]
