# Supplementary material for: Modeling Glial Contributions to Seizures and Epileptogenesis: Cation-Chloride Cotransporters in Drosophila melanogaster
Source: PLoS One. 2014 Jun 27;9(6):e101117. doi: 10.1371/journal.pone.0101117 (PMC4074161; doi:10.1371/journal.pone.0101117)
Supplement: Table S1 — The genotypes and sources of the Drosophila strains used in this study. (DOC) [file pone.0101117.s002.doc]

**Table S1. *Drosophila*** strains used in this study.

| **Referred to as** | **Genotype** | **Stock (source, ID#)** |
| --- | --- | --- |
| 078Y | *w*; P{GawB}078Y* | Bloomington, 30821 |
| 104Y | *w^*^;104Y-GAL4* | Tanouye, T161 |
| 109(2)80 | *y^1^ w^*^; P{GawB}109(2)80* | Bloomington, 8769 |
| *10XUAS-mCD8::GFP* | *w*; P{10XUAS-IVS-mCD8::GFP}su(Hw)attP5* | Bloomington, 32188 |
| 201Y | *w^1118^; P{GawB}Tab2^201Y^* | Bloomington, 4440 |
| 221 | *w^*^; Pin^1^/CyO; P{GawB}221w^-^* | Bloomington, 26259 |
| *40XUAS-mCD8::GFP* | *w*; P{40XUAS-IVS-mCD8::GFP}attP2* | Bloomington, 32195 |
| 43 | *w^*^; P{GawB}43* | Bloomington, 7148 |
| 477 | *w^*^; P{GawB}477/CyO* | Bloomington, 8737 |
| 4G | *w^*^; P{GawB}4G* | Bloomington, 6927 |
| 5-HT | *w^*^; P{GAL4-5-HTR1B.Y}2* | Bloomington, 27636 |
| A307 | *w^*^;A307-GAL4* | Tanouye, T061 |
| Act5C | *y^1^ w^*^; P{Act5C-GAL4-w}E1/CyO* | Bloomington, 25374 |
| Akh | *y^1^ w^*^; P{Akh-gal4.L}2/CyO, y^+^* | Bloomington, 25683 |
| alrm | *w^*^;alrm-GAL4 (#3)/CyO;Dr/TM3, Sb^1^, e* | Marc Freeman |
| c061 | *w^*^; P{GawB}c061* | Bloomington, 30845 |
| c17 | *w^*^; P{GAL4}c17/CyO* | Bloomington, 39690 |
| c21 | *P{GAL4-Eh.2.4}C21* | Bloomington, 6301 |
| c232 | *w^*^; P{GawB}Aph-4^c232^* | Bloomington, 30828 |
| c346 | *w^*^; P{GawB}c346* | Bloomington, 30831 |
| c507 | *w^*^; P{GawB}Aph-4^c507^* | Bloomington, 30840 |
| c632a | *w^*^; P{GawB}c632a* | Bloomington, 30846 |
| c739 | *y^1^ w^67c23^; P{GawB}Hr39^c739^* | Bloomington, 7362 |
| c772 | *w^*^;c772-GAL4/CyO* | Tanouye, T165 |
| Canton Special | Wild-type | Tanouye, T801 |
| Cha [1] | *w^*^; P{Cha-GAL4.7.4}19B P{UAS-GFP.S65T}Myo31DF^T2^* | Bloomington, 6793 |
| Cha [2] | *w^1118^; P{Cha-GAL4.7.4}19B/CyO, P{sevRas1.V12}FK1* | Bloomington, 6798 |
| CQ2 [1] | *y^1^ w^*^; P{CQ2-GAL4}O* | Bloomington, 7466 |
| CQ2 [2] | *y^1^ w^*^; P{CQ2-GAL4}H* | Bloomington, 7468 |
| CyO, Act-GFP | *w^*^; In(2LR)noc^4L^Sco^rv9R^, b^1^/CyO, P{ActGFP}JMR1* | Bloomington, 4533 |
| D42 | *w^*^; P{GawB}D42* | Bloomington, 8816 |
| DJ761 | w^1118^; P{GawB}DJ761 | Bloomington, 8185 |
| elav^C155^ | *P{GawB}elav^C155^* | Bloomington, 458 |
| *elav^C155^>mCD8::GFP* | *P{GawB}elav^C155^, P{UAS-mCD8::GFP.L}Ptp4E^LL4^, P{hsFLP}1, w^*^* | Bloomington, 5146 |
| exex | *w^*^; P{GawB}exex^GAL4^ P{lacW}wah^S009413^/TM3, P{GAL4-Kr.C}DC2, P{UAS-GFP.S65T}DC10, Sb^1^* | Bloomington, 32555 |
| Gad1 | *w^*^;Gad1-GAL4* | Tanouye, T152 |
| gcm | *y^1^ w^*^; P{GawB}gcm^rA87.C^/CyO* | Bloomington, 35541 |
| Gli | *w^*^;gliotactin-GAL4 (RL82-GAL4)* | Vanessa Auld (via Marc Freeman) |
| GMR | *w^1118^; P{GMR-GAL4.w^-^}2/CyO* | Bloomington, 9146 |
| Gr66a | *w^*^; P{Gr66a-GAL4.D}2; Gr93a^3^* | Bloomington, 28801 |
| He | *w^*^; P{He-GAL4.Z}85, P{UAS-GFP.nls}8* | Bloomington, 8700 |
| hsp-70 | *w^*^;hsp-70-GAL4/CyO* | Louise Parker |
| *kcc^Ad-4^/CyO, Act-GFP* | *dp^ov1^ cn^1^ bw^1^ kcc^Ad-4^/ CyO, P{ActGFP}JMR1* | This study |
| *kcc^Ad-4^/SM6a* | *dp^ov1^ cn^1^ bw^1^ kcc^Ad-4^/SM6a* | Bloomington, 5207 |
| *kcc^EY08304^/CyO* | *y^1^ w^67c23^; P{EPgy2}kcc^EY08304^/CyO* | Bloomington, 16887 |
| *kcc^EY08304^/CyO, Act-GFP* | *y^1^ w^67c23^; P{EPgy2}kcc^EY08304^/ CyO, P{ActGFP}JMR1* | This study |
| L(3)31-1 | *w^*^; P{GawB}l(3)31-1^31-1^/TM6C, Sb^1^ Tb^1^* | Bloomington, 5820 |
| Lsp2 | *y^1^ w^1118^; P{Lsp2-GAL4.H}3* | Bloomington, 6357 |
| MB247 | *w^*^;;MB247-GAL4* | Tanouye, T160 |
| moody | *w^*^;spg-GAL4 [B-1]* | Roland Bainton (via Marc Freeman), C/9/A |
| mz0709 | *w^*^;;mz0709-GAL4* | Kai Ito (via Marc Freeman) |
| Mz97 | *w^*^; P{GawB}Mz97 P{UAS-Stinger}2* | Bloomington, 9488 |
| nan | *w^*^; P{nan-GAL4.K}2; nan^dy5^* | Bloomington, 24903 |
| neur | *w^1118^; P{GawB}neur^GAL4-A101^ Kg^V^/TM3, Sb^1^* | Bloomington, 6393 |
| ninaE | *w^*^; P{ninaE-GAL4.D}2; P{UAS-41Q.HA}3* | Bloomington, 30540 |
| NP2222 | *w^*^;NP2222-GAL4/CyO* | Marc Freeman, C/10/D |
| nrv2 | *w*; P{nrv2-GAL4.S}3* | Bloomington, 6800 |
| OK107 | *w^*^; P{GawB}ey^OK107^/In(4)ci^D^, ci^D^ pan^ciD^ sv^spa-pol^* | Bloomington, 854 |
| OK6 | *w^*^;OK6-GAL4* | Grant Kauwe |
| Or83b | *w^*^;P{Orco-GAL4.W}11.17; TM2/TM6B, Tb^1^* | Bloomington, 26818 |
| pain | *w^*^; P{GawB}pain^GAL4^* | Bloomington, 27894 |
| pdf | *y^1^ w^*^; P{Pdf-GAL4.P2.4}2* | Bloomington, 6900 |
| ppk | *w^*^; P{ppk-GAL4.G}3* | Bloomington, 32079 |
| ppk [1] | *Df(1)pod1^Δ17^, w^1118^ pod1^Δ17^ P{FRT(w^hs^)}9-2/FM7c, P{GAL4-Kr.C}DC1, P{UAS-GFP.S65T}DC5, sn^+^; P{ppk-GAL4.G}2, P{UAS-mCD8::GFP.L}LL5/CyO* | Bloomington, 8749 |
| ppk [2] | *w^*^; P{ppk-GAL4.G}2* | Bloomington, 32078 |
| R1 | *P{rh1-GAL4}1; ry^506^* | Bloomington, 8688 |
| R7 | *w^*^; P{Pan-R7-GAL4}2/CyO* | Bloomington, 8603 |
| Rdl | *w^*^;;Rdl-GAL4-2-1(3)* | Tanouye, T352 |
| repo | *w^*^;;repo-GAL4/TM3, Sb^1^* | Kai Ito (via Marc Freeman) |
| sca | *y^1^ w^*^; P{GawB}sca^109-68^/CyO* | Bloomington, 6479 |
| SG18 | *w^*^; P{GawB}SG18.1* | Bloomington, 6405 |
| Sgs3 | *w^1118^; P{Sgs3-GAL4.PD}TP1* | Bloomington, 6870 |
| TH | *w^*^; P{ple-GAL4.F}3* | Bloomington, 8848 |
| tim | *y^1^ w^*^; P{GAL4-tim.E}62* | Bloomington, 7126 |
| tub-GAL80^ts^ [1] | *w^*^; sna^Sco^/CyO; P{tubP-GAL80^ts^}7* | Bloomington, 7018 |
| tub-GAL80^ts^ [2] | *w^*^; P{tubP-GAL80^ts^}20; TM2/TM6B, Tb^1^* | Bloomington, 7019 |
| UAS-kcc-RNAi-B | *y^1^ sc^*^ v^1^; P{TRiP.HMS01058}attP2* | Bloomington, 34584 |
| *UAS-kcc-RNAi-B,tubulin-GAL80^ts^[2* | *w^*^; P{tubP-GAL80^ts^}20; P{TRiP.HMS01058}attP2* | This study |
| UAS-kcc-RNAi-V | *P{KK107965}VIE-260B* | VDRC, 101742 |
| *UAS-kcc-RNAi-V,tubulin-GAL80^ts^[1]* | *w^*^; P{KK107965}VIE-260B; P{tubP-GAL80^ts^}7* | This study |
| UAS-ncc69-RNAi-B | *y^1^ v^1^; P{TRiP.JF03097}attP2* | Bloomington, 28682 |
| UAS-ncc69-RNAi-V | *w^1118^; P{GD14863}v30000* | VDRC, 30000 |
