# Supplementary material for: Modeling Glial Contributions to Seizures and Epileptogenesis: Cation-Chloride Cotransporters in Drosophila melanogaster
Source: PLoS One. 2014 Jun 27;9(6):e101117. doi: 10.1371/journal.pone.0101117 (PMC4074161; doi:10.1371/journal.pone.0101117)
Supplement: Table S2 — All results from the UAS-kcc-RNAi-V and UAS-kcc-RNAi-B screen for RNAi-induced behavioral seizure-like activity. (DOC) [file pone.0101117.s003.doc]

**Table S2. Candidate GAL4 screen for behavioral seizure-like activity caused by driving UAS-kcc-RNAi-V and UAS-kcc-RNAi-B.**

| **GAL4 driver** | **%BS paralysis with kcc-RNAi-V** | **%BS paralysis with kcc-RNAi-B** |
| --- | --- | --- |
| 078Y | 8 | Untested |
| 104Y | 0 | Untested |
| 109(2)80 | 1 | Untested |
| 201Y | 0 | 100 |
| 221 | 2 | Untested |
| 43 | 2 | Untested |
| 477 | 11 | Untested |
| 4G | 44 | Untested |
| 5-HT | 0 | Untested |
| A307 | 100 | Lethal |
| Act5C | Lethal | Lethal |
| Akh | Untested | 0 |
| alrm | 22 | 23 |
| c061 | 0 | Untested |
| c17 | 0 | Untested |
| c21 | 0 | Untested |
| c232 | 7 | Untested |
| c346 | 0 | Untested |
| c507 | 20 | Lethal |
| c632a | 3 | Untested |
| c739 | 0 | Untested |
| c772 | 0 | 100 |
| Cha [1] | 100 | Lethal |
| Cha [2] | 100 | Lethal |
| CQ2 [1] | 0 | Untested |
| CQ2 [2] | 0 | Untested |
| D42 | 97 | Untested |
| DJ761 | 0 | Untested |
| elav^C155^ | Lethal | Lethal |
| exex | 56 | Untested |
| Gad1 | 0 | Untested |
| gcm | Untested | 0 |
| Gli | 18 | 100 |
| GMR | Untested | 0 |
| Gr66a | 0 | Untested |
| He | Untested | 0 |
| L(3)31-1 | 100 | Untested |
| Lsp2 | Untested | 0 |
| MB247 | 0 | 100 |
| moody | 0 | 61 |
| mz0709 | 40 | 71 |
| Mz97 | 0 | Untested |
| nan | 2 | Untested |
| nrv2 | 0 | 66 |
| neur | 10 | Untested |
| ninaE | 0 | 0 |
| NP2222 | 31 | 100 |
| OK107 | 0 | 96 |
| OK6 | 0 | Lethal |
| Orco | 0 | Untested |
| pain | 0 | Untested |
| Pdf | 0 | Untested |
| ppk | 0 | Untested |
| ppk [1] | 1 | Untested |
| ppk [2] | 0 | Untested |
| R1 | 0 | Untested |
| R7 | 0 | Untested |
| Rdl | 0 | Untested |
| repo | 70 | 87 |
| sca | 42 | Untested |
| SG18 | 0 | Untested |
| Sgs3 | Untested | 0 |
| TH | 0 | Untested |
| tim | Untested | 0 |
